# Evaluation of *de novo* donor specific antibodies after kidney transplantation in the era of donor-derived cell-free DNA

**DOI:** 10.3389/fimmu.2024.1530065

**Published:** 2025-01-16

**Authors:** Yuan Tian, Lukas Frischknecht, Anna Mallone, Fabian Rössler, Thomas Schachtner, Jakob Nilsson

**Affiliations:** ^1^ Department of Immunology, University Hospital Zurich (USZ), Zurich, Switzerland; ^2^ Department of Surgery and Transplantation, University Hospital Zurich, Zurich, Switzerland; ^3^ Division of Nephrology, University Hospital Zurich, Zurich, Switzerland

**Keywords:** kidney transplantation, Dd-cfDNA, donor specific antibodies, DSA, mean fluorescence intensity, MFI

## Abstract

**Background:**

Donor-derived cell-free DNA (dd-cfDNA) is a promising non-invasive biomarker for detecting graft injury in solid organ transplant recipients. Elevated dd-cfDNA levels are strongly associated with rejection and graft injury, especially antibody-mediated rejection (ABMR). While *de novo* donor-specific antibodies (dnDSA) are crucial in ABMR, the relationship between dd-cfDNA levels and dnDSA features, such as DSA category, MFI and HLA target loci, remains unclear.

**Methods:**

We analyzed dd-cfDNA levels in 75 kidney transplant recipients who developed dnDSA post-transplant. dnDSA were categorized as “true”, “possible”, or “false” based on bead reactivity patterns and HLA typing. dd-cfDNA was assessed alongside dnDSA detection and sequential follow-up samples in a subgroup.

**Results:**

“True” dnDSA showed significantly higher dd-cfDNA levels compared to “possible” and “false” groups. None of the dd-cfDNA values in the “false” group exceeded 0.6%, and only a small fraction of the “possible” group had values slightly above 0.6%. dd-cfDNA levels were not significantly affected by dnDSA target loci or number. A strong correlation between cumulative dnDSA MFI and dd-cfDNA levels was observed, especially in patients with “true” HLA-DQ-directed dnDSA. Sequential dd-cfDNA analysis showed dynamic changes in 25% of patients, all from the “true” dnDSA group, which tended to align with shifts in cumulative MFI over time.

**Conclusion:**

These findings highlight the correlation between cumulative dnDSA MFI and dd-cfDNA levels, particularly in HLA-DQ-directed dnDSA, and suggest graft injury is dynamic in dnDSA-positive patients. Integrated monitoring of dnDSA and dd-cfDNA offers a promising non-invasive approach for assessing graft injury and alloimmunity, potentially enhancing post-transplant care.

## Introduction

Donor-derived cell-free DNA (dd-cfDNA) measurements in peripheral blood have emerged as a promising diagnostic tool for detecting post-transplantation graft injury in solid organ transplant recipients (1–12). Due to the relatively short half-life of cell free DNA (cfDNA) in the circulation, dd-cfDNA measurements provide a potential snapshot of graft health, where elevated dd-cfDNA values indicate the concurrent destruction and release of cfDNA from cells within the donor graft (13).

Several large studies have associated elevated dd-cfDNA values with processes that cause graft injury in the setting of kidney transplantation, as well as with rejection episodes (4–6, 8). This suggests that dd-cfDNA could serve as a key non-invasive biomarker for rejection monitoring, potentially in conjunction with single antigen bead (SAB) anti-HLA antibody monitoring to detect the possible development of *de novo* donor specific antibodies (dnDSA) (14, 15). Elevated dd-cfDNA levels have been observed in both biopsy-proven T cell mediated rejection (TCMR) and antibody mediated rejection (ABMR), with some studies suggesting that a dd-cfDNA increase is more pronounced and detectable at an earlier time point in ABMR before classical signs of kidney graft rejection, such as decreased kidney function, are evident (5, 16, 17).

Furthermore, elevated dd-cfDNA values have been strongly associated with various classifiers of graft injury through Molecular Microscope (MMDx) analysis of kidney graft biopsies (17, 18). Notably, the highest dd-cfDNA values correlated with early or fulminant ABMR, as indicated by elevated transcripts associated with these pathologies in the MMDx evaluation. Interestingly, in cases where MMDx biopsy analysis did not fully meet the criteria for a diagnosis of rejection or graft injury, increased dd-cfDNA values were still associated with increased transcripts linked to rejection and/or graft injury (3, 19). This suggests that elevated dd-cfDNA values may arise from early stages of rejection or graft-destructive pathologies not yet classified.

The development of dnDSA or the presence of pre-transplant DSA targeting donor HLA proteins is closely associated with ABMR and an increased risk of subsequent graft loss (20–26). Monitoring dnDSA development post-transplant allows for the early detection of alloimmune responses, potentially enabling interventions to mitigate associated graft injury and prevent graft loss. Factors associated with pre-transplant DSA or dnDSA, such as the relative antibody level or binding strength indicated by mean fluorescence intensity (MFI), or the specific HLA loci targeted by DSA, have been shown to strongly influence the risk of rejection and graft loss (21, 22, 27, 28). The appropriate designation of a post-transplant dnDSA is not trivial as the correct assignment is dependent on the ability to accurately elucidate HLA specificity which can be complicated by allele specificity as well as alpha chain dependency in the setting of HLA Class II specific anti-HLA antibodies. Furthermore, the SAB assay is associated with unspecific reactivity in part due to the presence of denatured HLA antigens (29). Taken together, this makes accurate dnDSA designation post-transplant challenging and the addition of further biomarkers associated with early graft injury could potentially help improve dnDSA evaluation. However, the relationship between dnDSA development and dd-cfDNA levels post-transplant, as an indicator of ongoing graft injury remains unclear. Previous studies have associated increased dd-cfDNA levels with the presence of dnDSA, and it has been suggested that elevation of dd-cfDNA may precede dnDSA development (8). Additionally, MFI values above 2500 has been associated with a higher dd-cfDNA, with dnDSA target DQ loci, the most frequently observed and exhibiting the highest MFI values (7, 30). However, the connection between dd-cfDNA levels and dnDSA classification, target loci (Class I and Class II) or MFI, has not been extensively studied.

To explore these critical questions in post-transplant monitoring, we investigated a cohort of patients who received a kidney transplant at the University Hospital in Zurich between 2008 and 2024. Our study aims to elucidate the relationship between dnDSA development and dd-cfDNA levels in kidney transplant recipients, with the goal of enhancing our understanding of these important biomarkers and their potential role in improving post-transplant monitoring strategies.

## Methods

### Patient population

The study included patients who underwent kidney transplantation at the University Hospital of Zürich (USZ) between January 2008 and February 2024 and were monitored post-transplant at the USZ. Only patients who developed a dnDSA after 2021 and had plasma samples available from October 2021 to June 2024 were included in this study. This limitation in inclusion is due to the reliance on EDTA plasma to perform the dd-cfDNA assay where this was only available in the post-transplant follow-up period after October 2021. The dataset comprises complete information from 75 patients who developed dnDSA within the specified time frame, including 137 plasma samples. Additional data include date of transplantation, as well as detailed data on dnDSA development including trajectories for patients where multiple SAB measurements were present. The study was approved by the local Ethical Committee in Zurich (BASEC 2018-01182).

### HLA typing and anti-HLA antibody analysis

DNA-based HLA typing was performed using blood samples with various technologies, including sequence-specific oligonucleotide (SSO), sequence-specific primer (SSP) and next-generation sequencing (NGS) technologies. In addition to the standard donor HLA typing, further typing was conducted to assess additional loci if the recipient developed anti-HLA antibodies targeting untyped HLA loci post-transplantation. Consequently, a complete virtual cross-match (vXM) for the post-transplant development of dnDSA was available for all patients included in this study.

Post-transplant HLA antibody screening followed the local protocol at the USZ and was carried out using Luminex single-antigen bead (SAB) technology (LABScreen Single Antigen; OneLambda). dnDSA monitoring occurred at specific intervals: 1, 3, 6, 12, 18 and 24 months post-transplant, and annually thereafter. Additional testing for anti-HLA antibodies was performed at the treating physician’s discretion in cases of suspected rejection, as well as for monitoring trajectories of detected dnDSA. The presence of dnDSAs was defined by detection of newly developed donor-specific antibodies targeting HLA-A, B, C, DR (including DRB3,4 and 5), DQ and DP, with a normalized mean fluorescence intensity (MFI) greater than 500.

### *De novo* DSA evaluation

The dnDSAs detected post-transplant through our automated virtual cross match (vXM) algorithm were subsequently reviewed individually by a transplantation immunology specialist in a blinded fashion. This process involved determining whether the antibody exhibited true donor specificity by analyzing the single bead reactivity pattern and comparing it to the donor’s HLA typing. Additionally, detected dnDSAs were examined for epitope specificity, particularly to identify alpha chain-binding antibodies related to the HLA-DQ and DP loci. The reactivity patterns were further compared to lot-specific reactivity data from non-immunized male samples, which are continuously monitored in the USZ transplant laboratory. This comparison helped in identifying lot-specific unspecific reactivity. This analysis also accounted for any reactivity observed in the SAB assay against the recipients’ own HLA antigens. Based on this thorough evaluation, the dnDSA reactivity was categorized into three distinct groups, as described in the results section. In patients with pre-transplant DSA, the development of dnDSA was defined as the emergence of a new DSA post-transplant.

### Dd-cfDNA analysis and calculation

dd-cfDNA analysis was performed using the One Lambda Devyser Accept cfDNA kit, following the manufacturer’s protocol. This method utilizes targeted sequencing of 50 indel markers to measure allele frequency. CfDNA samples were isolated from plasma by Maxwell RSC ccfDNA Plasma Kit (Promega). Subsequently, a two-step multiplex PCR was conducted. In the first PCR (PCR1), marker-specific primers were used to amplify the target regions to create an amplicon library. This library was then diluted and used in the second PCR (PCR2), where adapters and unique indices were incorporated to enable sequencing. The indexed libraries were pooled and purified according to the Devyser kit’s instructions, and sequencing was carried out with 2 x 75 cycles on an Illumina Miniseq NGS platform. Pre-transplant genotyping was performed to identify informative markers for detecting donor-derived cfDNA in post-transplant samples. The Advyser Solid organs software calculated the donor fraction based on homozygous and heterozygous informative markers, as described in the manufacturer’s protocol.

Based on our in-house experience and clinical expertise, we established 0.6% as the dd-cfDNA cut-off. Additionally, a change threshold of 0.5% was implemented to monitor significant shifts in dd-cfDNA levels over time. These thresholds were selected in consultation with clinical experts at our hospital to ensure alignment with our patient population and diagnostic needs.

### Statistical methods

Various statistical tests were employed to evaluate significance in this study. One-way analysis of variance (ANOVA) with Tukey *post-hoc* testing was used to compare dd-cfDNA percentages between multiple groups, while an independent t-test was applied to compare dd-cfDNA levels between two groups. The Mann-Whitney U Test was used for unpaired data to analyze differences in MFI values between two groups, given the non-parametric nature of the data. Spearman correlations were conducted to evaluate relationships between MFI levels and dd-cfDNA values.

All statistical analyses were performed using R (version 4.2.4) and RStudio (version 2023.12.1 + 402). The following packages were used: tidyverse (2.0.0), reshape2 (1.4.4), readxl (1.4.3), ggdist (3.3.2), ggrain (0.0.4), ggpp (0.5.8.1), ggbeeswarm (0.7.2), cowplot (1.1.1), ggrepel (0.9.3), webr (0.1.5). Statistical significance was evaluated using individual p-values, with a threshold of <0.05 considered statistically significant.

## Results

### Study population characteristics

**Figure 1:**
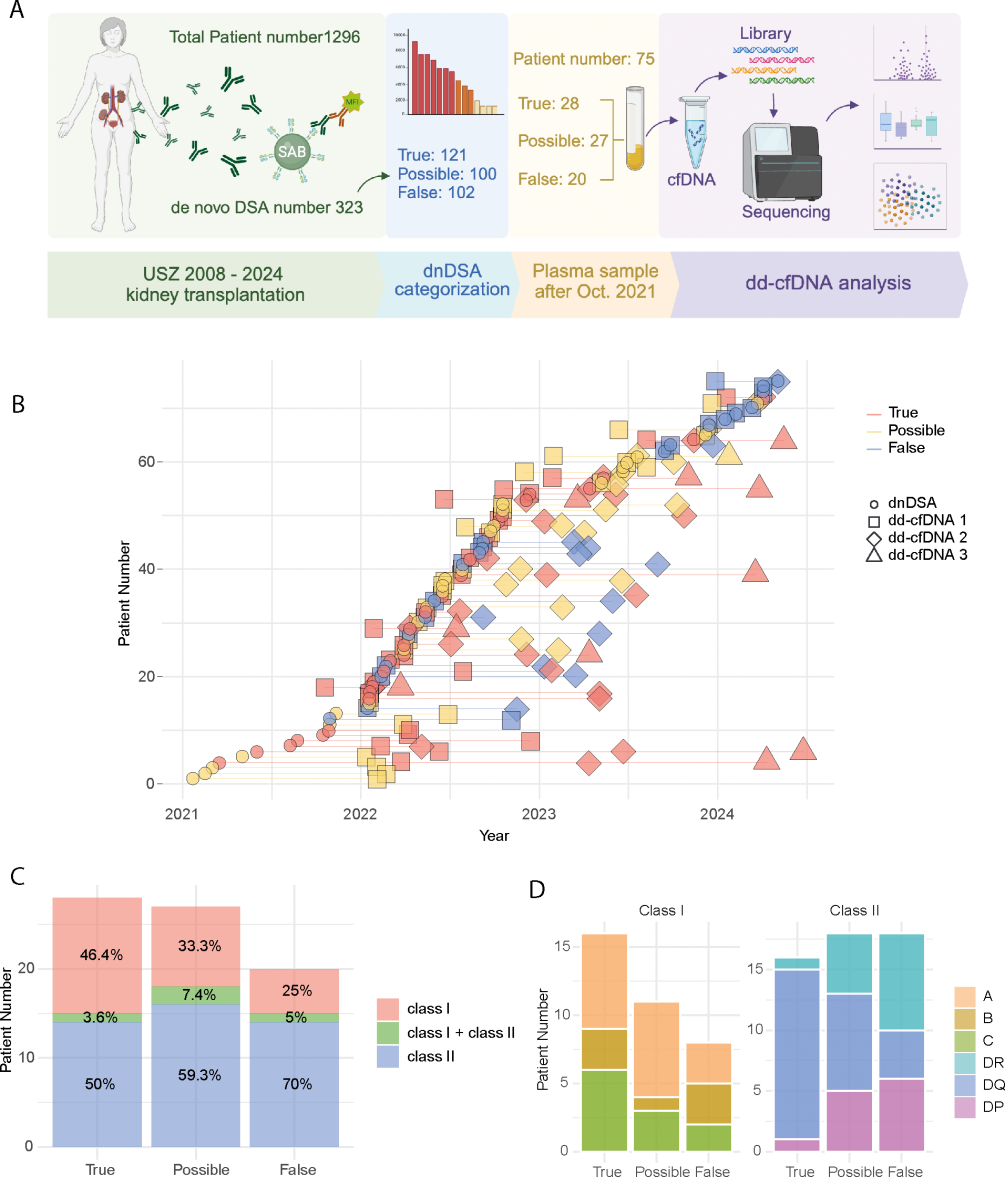
Summary of the study cohort. **(A)** Workflow of the study, showing patient numbers, dnDSA counts, and steps for dd-cfDNA analysis. Created with BioRender. **(B)** Timeline indicating for each patient dnDSA development date and plasma sample collection dates for dd-cfDNA detection. Lines connect multiple sequential samples from the same patient **(C)** Stacked area chart displaying the percentage of HLA class I and II specific dnDSAs, categorized into true, possible and false groups. **(D)** Stacked bar plot representing the number of patients with dnDSA targeting each HLA locus: class I (left) and class II (right).

An overview of patient inclusion and our analytical process is depicted in **Figure 1A**. The study considered patients for inclusion who received a kidney transplant at the University Hospital Zurich within the period from 2008 to 2024, and who were monitored post-transplant for the emergence of *de novo* donor-specific antibodies (dnDSA). The total cohort consisted of 1,296 individuals, with 808 male and 488 female kidney transplant recipients.

For this sub-study, only patients who developed a dnDSA during the post-transplant follow-up were considered for inclusion (n=323). Additionally, due to the reliance on stored EDTA plasma samples for the dd-cfDNA assay, only patients where a dnDSA was detected after October 2021 were included (n=75). For a portion of the included patients, multiple plasma samples were available for dd-cfDNA analysis. A detailed timeline of dnDSA detection and subsequent dd-cfDNA analysis for each included patient is shown in **Figure 1B**. Of the 75 patients, 59 (78.66%) had a dd-cfDNA sample collected on the same day as their dnDSA detection. In addition to these same-day samples, we included up to three total samples collected around the dnDSA detection date. If fewer than three additional samples were available, all available samples were included. **Supplementary Figure S1A** provides a timeline of the transplant date and dnDSA detection date for each patient, illustrating the interval between these key events. In total, 24 patients had only one sample, 40 patients had two samples, and 11 patients had three samples available for analysis.

### *De novo* DSA categorization

DnDSA were detected through the use of an automated virtual cross match (vXM) algorithm in our included patients and this was supplemented with additional HLA typing as needed to allow for a complete vXM. These automated results were then analyzed in a blinded fashion by a specialist in transplant immunology. The dnDSA were assessed for potential allele or alpha chain dependency and for association with known unspecific reactive beads, based on the study of lot specific anti-HLA antibody reactivity in non-immunized males.

Based on this detailed analysis, dnDSA-positive patients were divided into three groups: true dnDSA, possible dnDSA, and false dnDSA. The “true” group consisted of dnDSA with correct bead-based donor specificity without concerns for unspecific reactivity. The “possible” group included dnDSA where true dnDSA specificity could not be definitively confirmed or excluded. This uncertainty arose due to HLA typing results and the lack of a clear pattern of unspecific reactivity, where the reactivity was evaluated as not clearly representing a true dnDSA. The “false” group consisted of cases where “true” bead-based donor specificity could be excluded, as well as individuals where a clear unspecific reactivity was noted.

The HLA Class and locus targets of the detected dnDSA in the true, possible and false dnDSA groups are depicted in **Figures 1C, D**. False dnDSA were primarily directed at HLA Class II (70%), whereas true and possible dnDSA showed a more balanced HLA Class distribution (**Figure 1C**). Regarding individual HLA loci targets, there was a notable overrepresentation of HLA-DQ in the true dnDSA group compared to the possible and false groups. However, these differences are based on relatively few patients (n=75) and events (**Figure 1D**).

### True dnDSAs are associated with increased dd-cfDNA

**Figure 2:**
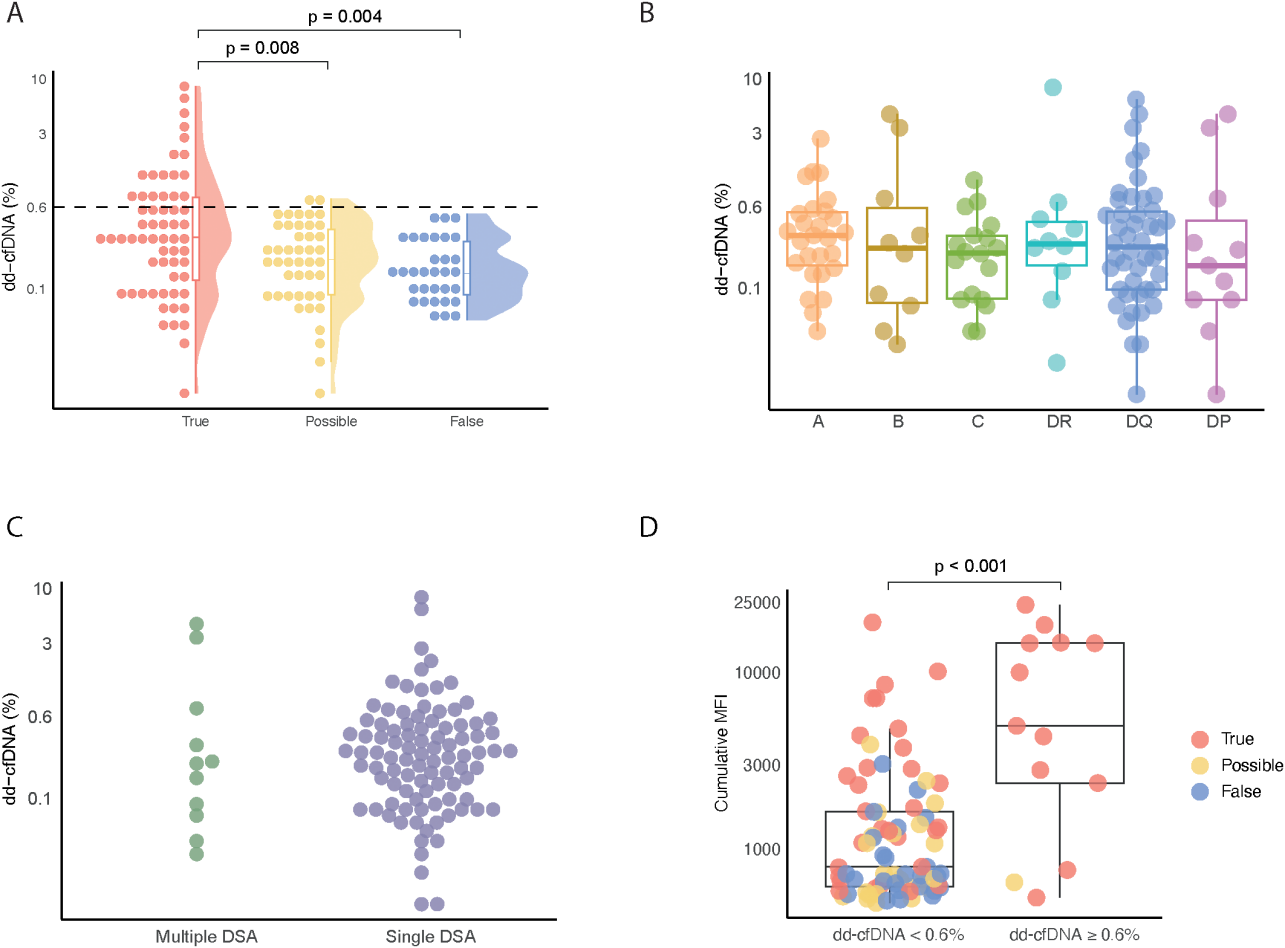
Comparison of dd-cfDNA levels across True, Possible, and Flase dnDSA groups. **(A)** dd-cfDNA values in plasma samples from patients with dnDSA, categorized into true, possible and false groups. **(B)** dd-cfDNA levels in plasma samples containing dnDSA against HLA-A, HLA-B, HLA-C, HLA-DR, HLA-DQ and HLA-DP. Single dnDSA in patients with multiple dnDSA are plotted individually. **(C)** Percentage of dd-cfDNA in plasma samples categorized into patients with dnDSA targeting multiple HLA loci versus a single HLA locus. **(D)** Cumulative MFI values in plasma samples grouped by a dd-cfDNA value of < or ≥ 0.6.

Next, we investigated the association between our dnDSA categorization and the first dd-cfDNA measurement performed at, or shortly after, dnDSA detection. Interestingly, the true dnDSA group showed significantly higher dd-cfDNA values compared to both the possible and false groups (**Figure 2A**). Notably, no dd-cfDNA values in the false dnDSA group exceeded 0.6%, and only two patients in the possible dnDSA group had values slightly above 0.6%. In total, none of the dd-cfDNA values in the possible and false groups exceeded 0.8% in our study (**Figure 2A**). **Supplementary Figure S1B** shows the same comparison using only the first sample from each group, which also demonstrated significant differences, although less pronounced.

We next focused on the true and possible dnDSA groups to explore how dnDSA target HLA loci related to dd-cfDNA levels. As shown in **Figure 2B**, we did not observe any marked differences relating to specific target HLA loci. It is important to note that this analysis was based on the association of single individual dnDSA with concurrent dd-cfDNA levels in patients where multiple dnDSA were detected.

To determine if there were differences in dd-cfDNA levels between patients with multiple or single dnDSA, we compared these two groups. As shown in **Figure 2C**, no marked differences were found in this analysis.

Given that a high cumulative DSA MFI has been strongly associated with ABMR development and graft loss, we decided to investigated the relationship between cumulative MFI and dd-cfDNA in our three dnDSA groups (true, possible and false) and using our previously suggested cut-off of 0.6%. Interestingly, a dd-cfDNA level ≥0.6% was associated with a significantly higher cumulative MFI as compared to a dd-cfDNA level <0.6% (**Figure 2D**).

### Cumulative true dnDSA MFI is tightly associated with dd-cfDNA levels

**Figure 3:**
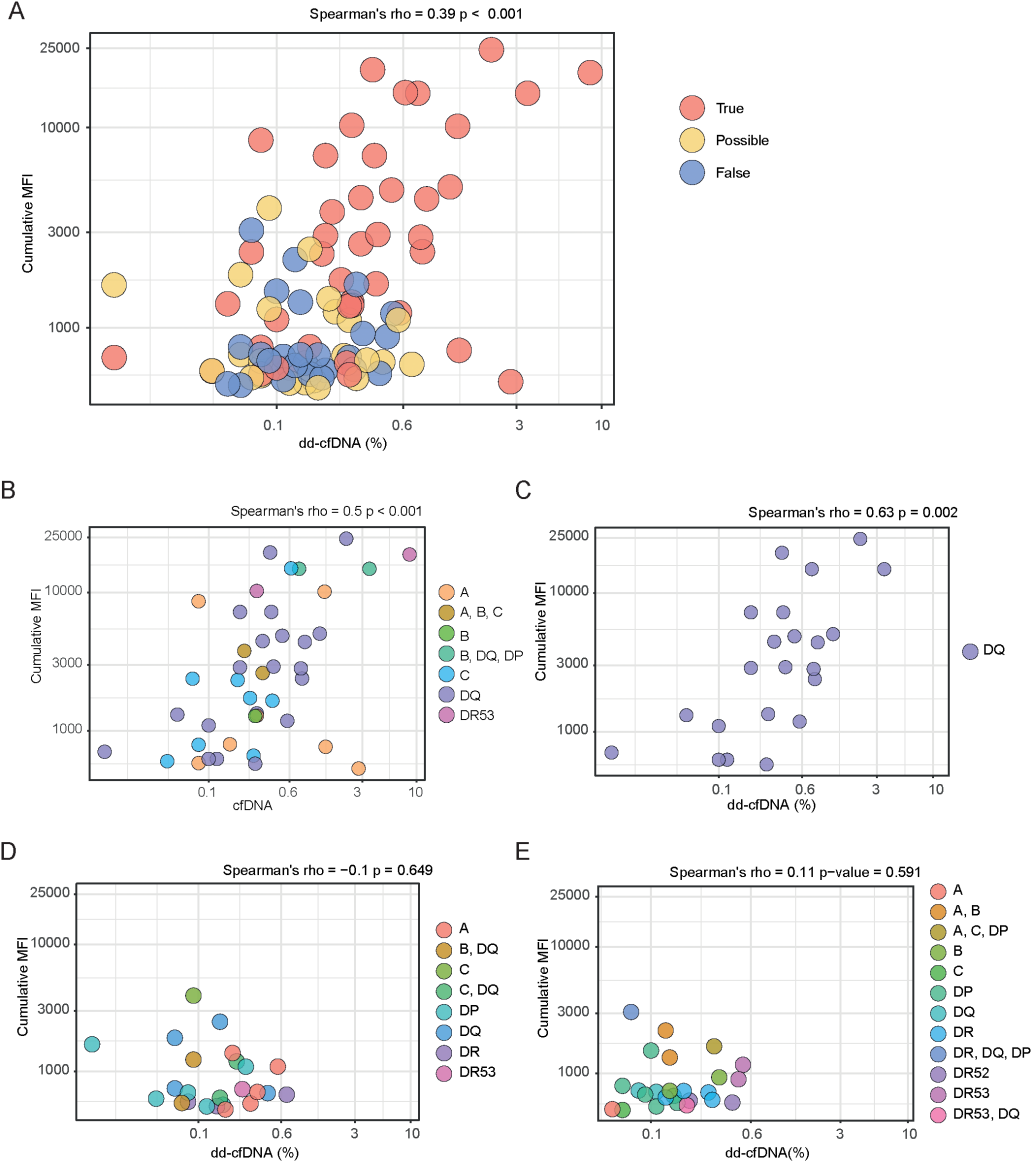
Spearman correlation between dd-cfDNA and dnDSA MFI in patient samples from True, Possible and False groups. **(A)** Scatter plot showing Spearman’s rank correlation between dd-cfDNA percentage and dnDSA MFI values across all samples, colored according to dnDSA category. **(B)** Correlation between dd-cfDNA level and dnDSA MFI value in the True group, with points colored by dnDSA single or combination target HLA loci. **(C)** Scatter plot depicting correlation between dd-cfDNA levels and dnDSA MFI values in samples from individuals with true dnDSA targeting the HLA-DQ locus. **(D, E)** Scatter plots of Spearman’s rank correlation between dd-cfDNA levels and dnDSA MFI values, colored by dnDSA targets from individuals in the possible **(D)** or false **(E)** dnDSA group. Panels **(A-E)** represent all measurement samples rather than individual patients.

Based on our finding that dd-cfDNA values ≥0.6% were associated with higher cumulative dnDSA MFI values, we next performed a Spearman correlation of cumulative dnDSA MFI and dd-cfDNA across all of the dnDSA groups (**Figure 3A**). This analysis revealed a significant correlation with a rho of 0.39, which looked to be primarily driven by observations in the true dnDSA group (**Figure 3A**).

Given this finding, we focused on the true dnDSA group and analyzed how individual dnDSA HLA target loci profiles, along with cumulative MFI, correlated with dd-cfDNA levels (**Figure 3B**). This analysis again showed a significant correlation, with a rho of 0.5. It was evident that individuals with single dnDSA targeting HLA-DQ, as well as those with multiple dnDSA containing HLA-DQ, were prominent in driving this correlation, reflecting the dominance of HLA-DQ as a target in our true dnDSA group (**Figure 3B**). To explore this further, we next analyzed only individuals with dnDSA containing HLA-DQ in the “true” group and examined the relationship between cumulative MFI and dd-cfDNA levels (**Figure 3C**). This analysis yielded a significant correlation with a rho of 0.63, despite being based on relatively few events. We next analyzed the association between cumulative MFI and dd-cfDNA levels in our “possible” and “false” dnDSA groups (**Figures 3D, E**). These analyses did not show any significant correlations, either for the possible (**Figure 3D**) or the false (**Figure 3E**) dnDSA group, further strengthening the validity of our dnDSA categorization.

### Dynamic in dd-cfDNA levels is associated with true dnDSA development

**Figure 4:**
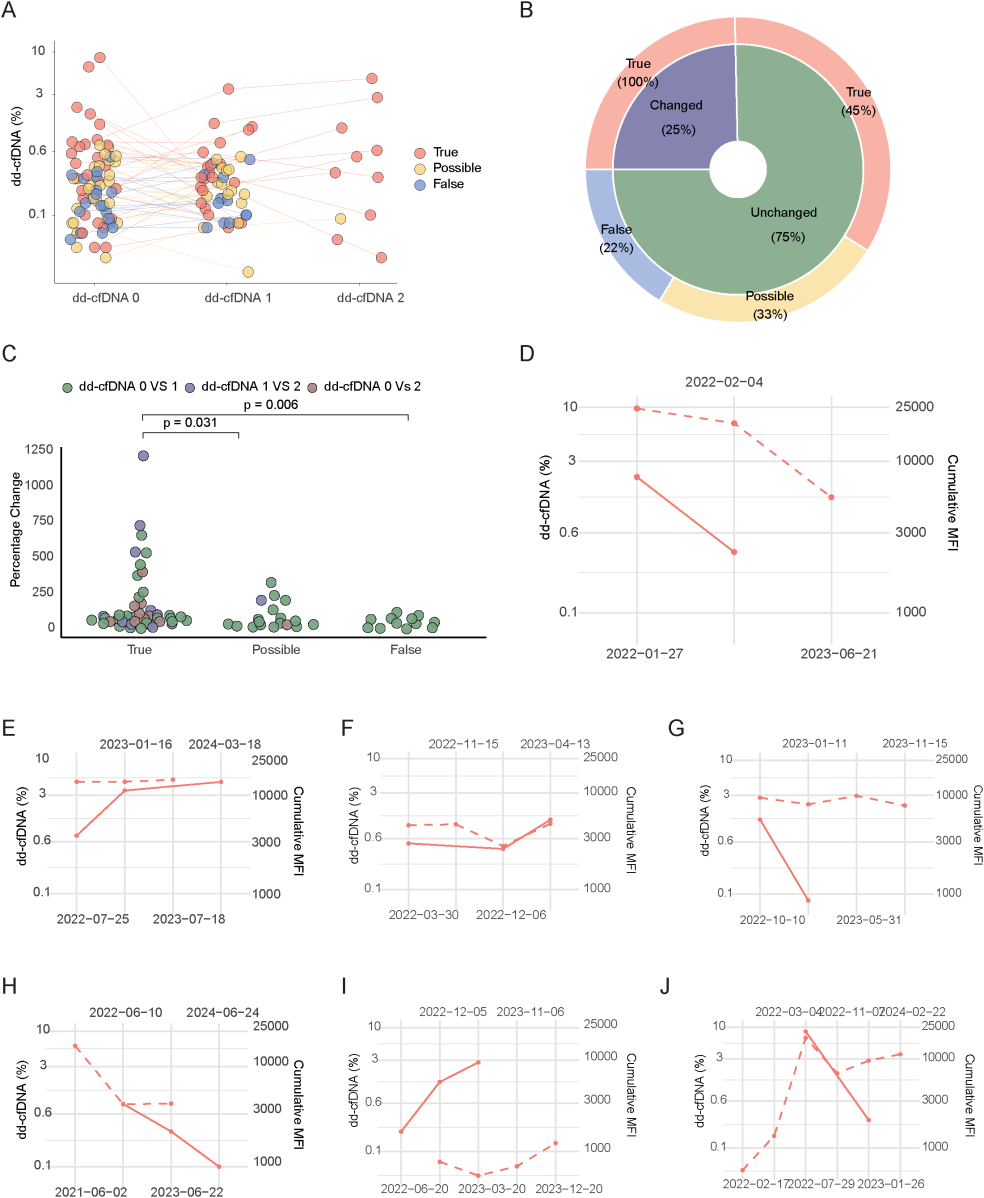
Trajectory of dd-cfDNA and MFI values overtime. **(A)** Trajectories of dd-cfDNA levels for each patient, colored according to True, Possible and False dnDSA categories. **(B)** Pie chart showing the proportion of patients with dd-cfDNA changes greater than 0.5% absolute percentage points between two time points, in the different dnDSA categories. **(C)** Percentage of dd-cfDNA level changes across True, Possible and False groups, colored by the timepoint comparisons. **(D-J)** Individual patient dd-cfDNA and MFI value trajectories from a subgroup showing dd-cfDNA dynamic and having more than two values from both dd-cfDNA and MFI. Each plot represents a single patient. The dotted line represents MFI and the filled line dd-cfDNA level.

For a subgroup of our included patients, we had access to multiple samples post-dnDSA detection, allowing us to perform up to three sequential dd-cfDNA analyses. This enabled us to analyze the dynamics of dd-cfDNA levels over time within this subgroup. An overview of dd-cfDNA levels over time for all included patients is presented in **Figure 4A**. In this analysis, we observed variations in the distribution of samples across patients within each annotation group. Specifically, among True patients, 5 had one sample, 13 had two samples, and 10 had three samples. For the Possible group, 11 patients had one sample, 15 had two samples, and 1 had three samples. In the False group, 8 patients had one sample and 12 had two samples. To categorize the dynamics of dd-cfDNA samples over time, we used a percentage point change of >0.5% between measurements as an indicator of a likely relevant change in dd-cfDNA amounts. Based on this criterion, we divided our patients with multiple dd-cfDNA measurements into two groups: those showing a ±0.5% point dynamic between measurements and those who did not. We then analyzed how this dynamic related to our dnDSA categories. Interestingly, as shown in **Figure 4B**, only 25% of the patients with multiple dd-cfDNA measurements exhibited a marked change in their levels, and all of whom were in the true dnDSA category.

To further evaluate this without relying on an appraised cut-off, we quantified the percentage change between measurements and plotted our patients with multiple dd-cfDNA measurements according to their dnDSA category. As shown in **Figure 4C**, patients in the true dnDSA category displayed a significantly higher change in dd-cfDNA values compared to those in the possible and false categories. Given the strong correlation previously noted between dd-cfDNA values and cumulative dnDSA MFI, we sought to investigate how sequential dd-cfDNA levels compared to cumulative MFI values in patients where such data were available. Due to the limited number of patients with both multiple dynamic dd-cfDNA values and multiple dnDSA MFI values over time, we individually plotted the seven patients who met these criteria. Interestingly, as shown in **Figure 4D-J**, there was a tendency for directional similarities in several patients regarding the dynamics of dd-cfDNA levels and dnDSA MFI.

## Discussion

The analysis of dd-cfDNA is emerging as a valuable non-invasive tool in post-transplant monitoring and it has the potential to improve solid organ transplant outcomes by facilitating an estimation of concurrent graft injury (1–12). This can aid in the early detection of pathologies leading to graft loss and allow for longitudinal monitoring of injury kinetics, especially during interventions aimed at addressing these pathologies.

The development of dnDSA post-transplant has been established as an important possible early marker of alloimmunity, increasing risk for rejection and graft loss (23, 31). Many transplant centers, therefore, monitor patients for dnDSA development (21, 32). When dnDSA are detected, it often leads to graft biopsies or interventions such as increased immunosuppression to counteract the detected alloimmune response (33). However, evaluating dnDSA in clinical practice is challenging, since unspecific reactivity and vastly different trajectories regarding graft loss and rejection in patients who develop dnDSA make it difficult to determine whether invasive diagnostic tests, such as graft biopsies, or interventions, like increased immunosuppression, should be initiated (34).

The combination of dnDSA measurements with dd-cfDNA analysis could potentially improve this evaluation, this is however an emerging field, and there is limited data that considers important dnDSA-associated factors such as specificity, MFI, and HLA targets (7, 9). Our study aims to enhance understanding of how dd-cfDNA values relate to key anti-HLA antibody features in a cohort of patients who developed dnDSA post-transplant and we decided to investigate dd-cfDNA values in relation to three designated dnDSA categories currently used clinically in our transplant center.

Interestingly, significantly elevated dd-cfDNA values were only observed in the group with true dnDSA, suggesting that dd-cfDNA levels above 0.6% might indicate elevated graft injury, at least in our limited analysis of patients with dnDSA development. This finding aligns with previous larger studies using biopsy-proven rejection as an endpoint, further highlighting the association of true dnDSA with graft injury (1, 4). Additionally, our findings support the relevance of our clinical dnDSA categorization and suggest that dd-cfDNA measurement can perhaps further refine the assessment of dnDSA in clinical practice. Our analysis of dd-cfDNA levels in relation to key dnDSA features showed that MFI was the main predictor of the extent of graft injury, as reflected by elevated dd-cfDNA. This is consistent with prior research showing that high dd-cfDNA levels are linked to a greater chance of dnDSA MFI values above 2500 (7). In contrast, neither HLA target loci nor the presence of multiple versus single dnDSA significantly impacted dd-cfDNA levels in our study. It is important to note, however, that our study included relatively few patients and a limited number of true and possible dnDSA cases, particularly for less common targets such as HLA-DP. Larger studies with more diverse patient populations are necessary to validate these initial results and further explore the thresholds of dd-cfDNA associated with significant graft injury.

The robust correlation between dnDSA MFI and dd-cfDNA levels was strongest in patients with dnDSA directed against HLA-DQ. This may be due to the predominance of HLA-DQ dnDSA in our true dnDSA group, but it could also reflect the particularly detrimental effect of DSA targeting HLA-DQ on transplant outcomes (22, 35). Our finding of a strong association between HLA-DQ dnDSA MFI and dd-cfDNA builds on previous studies that have linked high MFI anti-HLA DQ dnDSA and graft loss (36). We demonstrate that these antibodies are not only associated with graft loss but also with severe graft injury already at dnDSA detection, as indicated by elevated dd-cfDNA levels, which closely correspond to the concurrent MFI of the dnDSA. Our study is one of the first to establish a strong correlation between dnDSA MFI and dd-cfDNA levels, particularly in patients with HLA-DQ-directed dnDSA. This novel finding underscores the potential of combining these two biomarkers to offer a more refined and non-invasive approach to detecting and monitoring graft injury in post-transplant care. Importantly, no correlation was found between cumulative dnDSA MFI and dd-cfDNA levels in the possible or false dnDSA groups, which reinforces the clinical validity of our dnDSA classification and suggests that potential additional invasive diagnostic approaches in such cases may be omitted.

Sequential measurement of dd-cfDNA also allowed us to evaluate how graft injury develops over time, suggesting that it could be used to monitor therapeutic interventions. In a limited subgroup, we explored how dd-cfDNA dynamics related to dnDSA categories and dnDSA MFI trajectories. Only patients with “true” dnDSA showed dynamic changes in dd-cfDNA levels. This is likely related to the process of antibody-mediated rejection and interventions aimed at mitigating these processes (37). The primary limitation of this analysis is the small number of patients in the Possible and False groups with multiple samples, which restricts the ability to draw robust conclusions about dd-cfDNA dynamics in these categories. While the study included a small cohort of patients with multiple dd-cfDNA and dnDSA measurements, the findings highlight the potential of future research focusing on dnDSA and dd-cfDNA trajectories to improve our understanding of how combined monitoring could influence treatment decisions.

In summary, our data strongly link dnDSA classification and dnDSA MFI with concurrent graft injury, as measured by dd-cfDNA. Furthermore, our findings suggest that dynamic changes in dd-cfDNA levels are associated with clinically relevant dnDSA. While this study primarily focused on the relationship between dnDSA and dd-cfDNA, future research should explore additional factors, such as mixed rejection phenotypes involving cell-mediated injury, to provide a more comprehensive understanding of graft injury mechanisms. Larger cohorts with detailed clinical data, including biopsy results and kidney function, could further validate these associations and provide insights into how dd-cfDNA and dnDSA monitoring can support personalized immunosuppressive management, ultimately improving post-transplant outcomes.

## Data Availability

The original contributions presented in the study are included in the article/**Supplementary Material**. Further inquiries can be directed to the corresponding author.
