# Supplementary figures and images for: Evaluation of de novo donor specific antibodies after kidney transplantation in the era of donor-derived cell-free DNA
Source: Front Immunol. 2025 Jan 16;15:1530065. doi: 10.3389/fimmu.2024.1530065 (PMC11779610; doi:10.3389/fimmu.2024.1530065)

supplementary figure 1

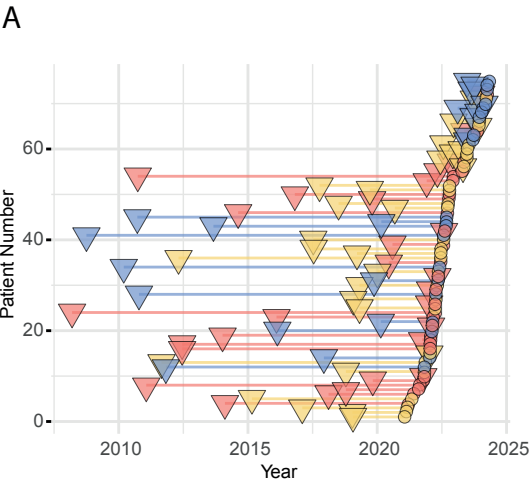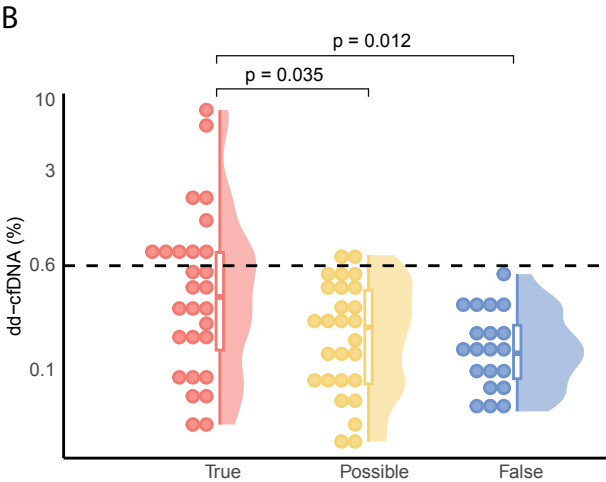

Supplement: Supplementary file 1 [file DataSheet1.pdf]
